# Founder pathogenic variants in colorectal neoplasia susceptibility genes in Ashkenazi Jews undergoing colonoscopy

**DOI:** 10.1038/s44276-024-00045-x

**Published:** 2024-03-05

**Authors:** Thibaut Matis, Celine Domecq, Nancy Hamel, Ester Castellsagué, Adriana Lopez-Doriga, Stephen Marotta, Peter Zauber, William D. Foulkes

**Affiliations:** 1 Cancer Genetics Unit, Institut Bergonié, Bordeaux, France; 2 BRIC (BoRdeaux Institute of onCology), UMR1312, INSERM, Univ. Bordeaux, F-33000 Bordeaux, France; 3 Cancer Research Program, Research Institute of the McGill University Health Centre, Montreal, QC H4A 3J1 Canada; 4 Department of Human Genetics, McGill University, Montreal, QC Canada; 5 Cancer Axis, Lady Davis Institute, Jewish General Hospital, Montreal, QC H3T 1E2 Canada; 6 Unit of Bioinformatics for Precision Oncology, Catalan Institute of Oncology (ICO), L’Hospitalet de Llobregat, Barcelona, Spain; 7 Cooperman Barnabas Medical Center, Livingston, NJ USA; 8 Present Address: 34 Cervantes St, Sant Just Desvern, Barcelona, Spain

## Abstract

**Background:**

Colorectal neoplasia is one of the most common tumors affecting Western populations.

**Methods:**

In this study we used a custom amplicon sequencing platform and an in-house bioinformatic pipeline to study constitutional DNA from two different case series of Ashkenazi Jews undergoing colonoscopy (*n* = 765). The first series all had pathologically confirmed colorectal adenomas and/or carcinoma. The second series consisted of persons who had undergone a colonoscopy within the five years prior to ascertainment, regardless of findings. Ninety-one percent of all patients were asymptomatic at the time of colonoscopy.

**Results:**

In the first group (*n* = 438), we identified 65 founder variants (56 in *APC*, 2 in *GREM1*, 3 in *MSH2* and 4 in *BLM*). In the second group (*n* = 327), the findings were 30, nothing, 1 and 1, respectively, as well as 2 *MSH6* variants.

**Conclusions:**

Overall, we found that 10 to 15% of Ashkenazi Jewish persons undergoing colonoscopy harbor variants of interest in colorectal and/or polyposis predisposition. This includes pathogenic variants in *MSH6*, which is associated with colorectal cancer but not with polyposis. We identified no pathogenic variants in more recently discovered polyposis predisposition genes (*POLE*, *POLD1* or *NTHL1*), rendering the presence of such founder variants rare.

## Introduction

Colorectal cancer (CRC) is a leading cause of morbidity and mortality in occidental countries [1]. Genetic predisposition to CRC due to germline pathogenic variants (GPVs) in cancer susceptibility genes has been implicated in 2–8% of all CRCs [2]. The most frequent CRC predisposition syndrome, Lynch syndrome, is caused by GPVs in one of several mismatch repair (MMR) genes, *MLH1*, *MSH2*, *MSH6* and *PMS2*, and *EPCAM* [3]. Hereditary polyposis due to GPVs in *APC*, *MUTYH*, *POLE*, *POLD1*, and more recently in *NTHL1* and *MSH3* [3–7], also contribute to CRC predisposition through increased adenoma prevalence and subsequent malignant transformation.

Ashkenazi Jews (AJ) are a recognized population group identified as carrying founder GPVs predisposing to several diseases, including CRC. The first CRC-associated alteration detected in this population was *APC* c.3920 T > A ; p.(Ile1307Leu, often referred to as *APC* I1307K) which at the nucleotide level changes an AAATAAAA track to an unstable AAAAAAAA track [8]. This phenomenon increases the replication error rate, leading to somatic mutations and conferring an increased CRC risk [9]. Additional mutations known to be involved in AJ CRC predisposition include the c.1906G>C mutation in *MSH2* [10, 11], c.3959_3962delCAAG, c.3956_3959delAAGC and c.3984_3987dupGTCA [12] in *MSH6*, and a *GREM1* 40 kb upstream duplication [13]. The role of *BLM*^*ash*^ c.2207_2212delinsTAGATTC variant [14] in CRC susceptibility is more controversial [15–17], so we thought it appropriate to include the variant as part of the panel for this large series.

Several different approaches have been reported as part of the effort to identify germline variants that are associated with an increased risk for the development of CRC. DNA from patients with known polyposis and/or CRC may be broadly screened for genetic changes and compared to samples from patients without CRC tumors. Subsequently, an identified variant might be assessed in a series of patients with CRC, with or without a control group. Populations studied are often heterogenous, and there are few studies limited to just the AJ population. Such studies are also usually limited to one, or just a few, of the putative pathogenic variants.

While several founder GPVs in classic CRC genes are well known in the Ashkenazi population, there is little information in the AJ regarding potentially pathogenic variants in cancer susceptibility genes more recently described in the general population (e.g. *POLE, POLD1*, and *NTHL1*). In this study, we aimed to assess the prevalence of AJ founder GPVs associated with colorectal polyps/CRC. To this end, we genotyped known AJ founder GPVs and sequenced targeted regions of more recently identified tumor susceptibility genes in the AJ population utilizing a large case series of AJ individuals known to have had tumors and using a similar, smaller, population clinically evaluated and determined to be free of such tumors. All patients were solicited through community clinics and not through referral to specialized centers.

## Method

### Patient selection & samples recording

765 DNA samples were collected from blood or Formalin-Fixed Paraffin-Embedded (FFPE) tissues from patients representing 765 AJ probands with CRC and polyps (*n* = 103), polyps (*n* = 497) or unaffected (*n* = 165) (Table 1). We also recorded familial information on all first-degree relatives (Table 1). Patients were accrued over several years between 2004 and 2015 and previously published in part [11, 18, 19]. Here, we combine these sequential studies into two series: CPC-series (colorectal polyp & cancer series) and CO-series (colonoscopy series). These three previous studies were concerned with the evaluation of the frequency of three founder GPV: *APC p.I1307K*, *MSH2 c.1906G* > *C* and the *BLM*^*ash*^ variant [11, 18, 19]. The first series (CPC-series) consisted of 438 individuals who had pathologically confirmed colorectal adenomas and/or carcinoma and were ascertained through pathology records.

**Table 1:** Population demography with personal and family history of cancer.

		Number	%
Source of constitutional DNA (*n* = 765			
- Adenoma		200	26,1%
- Blood		259	33,9%
- Tumor		3	0,4%
- Hyperplasia		53	6,9%
- Normal Mucosa		249	32,5%
- Other		1	0,1%
Sex (*n* = 765)			
- Female		323	42,2%
- Male		442	57,8%
Age at diagnosis			
Mean		66,7	
Median		69	
Personal history (*n* = 765)			
	- Polyps only	492	64,3%
	- Polyps & Colon Cancer	85	11,1%
	- Polyps & Rectal Cancer	11	1,4%
	- Polyps & Metachronous Colon Cancer	5	0,7%
	- Polyps & Rectal & Colon Cancers	2	0,3%
	- Polyps & Desmoid tumor	1	0,1%
	- Healthy – no colorectal tumors	165	21,6%
	- Unknown – could not verify colon status	4	0,5%
Familial history (first degree relative) (*n* = 759)			
	- Adenoma	5	0,7%
	- Colon Cancer	265	34,9%
	- Rectal Cancer	20	2,6%
	- Bladder Cancer	16	2,1%
	- Brain Cancer	10	1,3%
	- Breast Cancer	120	15,8%
	- Endometrial Cancer	20	2,6%
	- Esophagus Cancer	5	0,7%
	- Head and neck Cancer	4	0,5%
	- Kidney Cancer	34	4,5%
	- Leukemia	21	2,8%
	- Liver Cancer	9	1,2%
	- Lung Cancer	53	7,0%
	- Lymphoma	30	4,0%
	- Melanoma	19	2,5%
	- Myeloma	10	1,3%
	- Other	17	2,2%
	- Ovarian Cancer	9	1,2%
	- Pancreas Cancer	21	2,8%
	- Prostate Cancer	60	7,9%
	- Stomach Cancer	28	3,7%
	- Thyroid Cancer	7	0,9%

The second series (CO-series) consisted of 327 individuals who had undergone a colonoscopy up to five years prior to being recruited into the study and were ascertained through referrals from physicians. Patients were included in the CO-series regardless of pathology findings. Presence / absence of findings defined whether a participant is a case or control. Patients with a neoplasm became CO-patients. A neoplasm was considered present if at any time in their past a colonoscopy had revealed a tubular, tubulovillous, or villous adenoma; or a colorectal cancer (*n* = 162; polyps only: 135 (41.3%); cancer + polyps: 27 (8.3%)). Patients with no pathological findings became CO-controls (*n* = 165).

This series is composed of 162 affected persons: CO-patient series (CO-p) and 165 unaffected controls (CO-control). One patient was analyzed in both studies, several years apart, and we did not realize that she had been studied earlier. In the current study we considered her as one person. All patients were of self-reported AJ background and of northern European extraction, and they all resided in northern New Jersey. Sixty-four percent of all patients were recorded to have at least colorectal polyps, and nearly 34% have a familial history of colon cancer.

With the permission of the patients’ colonoscopists, patients were contacted to verify Ashkenazi ancestry and then invited to participate in a study designed to assay for germline variations in genes associated with colorectal tumors. Informed consent was obtained in writing from each participant. Personal and family history was obtained by telephone. The Institutional Review Board of the Saint Barnabas Medical Center (known as Cooperman Barnabas Medical Center since 2022) approved the studies (IRB study number: 03-41). Endoscopic findings were verified by reference to medical charts and pathology computerized records. All pathological slides were reviewed by one clinical pathologist. All information was stored in an independent computer only accessed by one study coordinator.

Right-sided lesions were defined as those removed from the cecum, ascending, and transverse colon segments. Left-sided lesions were those removed from the descending, sigmoid and rectal segments. In the course of the two studies, samples of DNA extracted from either pathological tissue or peripheral blood white cells were evaluated for known or putative AJ founder alleles, as discussed above [11, 18, 19]. We also looked for the *BLM*^*ash*^ variant. After the completion of the two studies, aliquots of DNA from all patients were shared with the research laboratory at the Research Institute of McGill University Hospital Center (RI MUHC) for a more comprehensive evaluation. This additional study was also approved by the Saint Barnabas Medical Center Institutional Review Board, as well as by the MUHC REB in Montreal (2018-3947).

### Sequencing

Regions analyzed include known/putative founder variants in, or selected exons/domains of, the following genes: *GREM1, BLM*, *APC*, *MSH2, MSH6*, *POLE*, *POLD1*, and *NTHL1* based on the GRCh37/hg19 genome reference. Only loci encompassing a founder mutation hotspot known to be involved in AJ were targeted in *GREM1* [20], *BLM*, *APC* [11], *MSH6* [12], *MSH2* [10]: *GREM1* 40 kb upstream duplication, *BLM* c.2207_2212delinsTAGATTC; p.(Tyr736Leufs*5), *APC* c.3920 T > A; p.(Ile1307Lys), *MSH6* c.3959_3962delCAAG; p.(Ala1320Glufs*6) and *MSH6* c.3984_3987dup; p.(Leu1330Valfs*12), *MSH2* c.1906G>C; p.(Ala636Pro). For *POLE* and *POLD1*, we targeted the exonuclease domain, essential for the maintenance of replication and described to be a hotspot region for germline colorectal cancer [5]: *POLE* exonuclease domains only: residues p268-471 and *POLD1* exonuclease domains only: residues p.304-526. The entire coding sequence and intron/exon boundaries were screened for *NTHL1* (6 exons, 1067 bp + intron/exon boundaries) and *MSH3* (24 exons, 4092 bp + intron/exon boundaries) for variant discovery.

We used a custom-designed microfluidics array chip (Standard Biotools, previously Fluidigm Corporation, South San Francisco, California) to amplify targeted sequences flanking known AJ CRC founder mutations, as well as covering the regions of interest within newly associated genes. To improve cost efficiency, samples were pooled (2 to 3 samples per pool) to reduce the number of chips necessary for capture before PCR amplification. Moreover, samples were pooled by quality to favor equal representation of each sample by PCR (i.e. DNA from blood samples together, or from good quality formalin fixed paraffin embedded (FFPE) tissues together). Thus, 285 pools were analysed. Sequencing was performed on an Illumina MiSeq paired-end 250 bp for amplicons designed accordingly (near 250 bp in size). Regions where amplicons failed for many samples were redesigned in the 150 bp range and re-sequenced on an Illumina MiSeq paired-end 150 bp run. We were able to obtain 1000x of coverage per sample. Reads were aligned using the BWA-MEM algorithm [21]. Resulting alignments were processed by Picard tools (http://broadinstitute.github.io/picard/); reads with a quality <30 were filtered out, and the remaining reads were sorted and compressed (Supplementary File 1). Some regions were re-sequenced to improve coverage when needed. We set up a variant calling filtering strategy by using the *APC* gene I1307K variant as a control since this variant had been previously assessed in most of the samples analyzed [19]. To obtain the highest sensitivity possible, we used a minimum frequency of 4% or a minimum coverage of 15x to identify a variant. Variant calling was accomplished using VarScan [22] and ANNOVAR [23]. Sanger sequencing was used to validate the presence of variants identified by NGS in the original individual samples and to identify the specific mutation carrier(s) within each sample pool.

### Filtering analysis for genes without known founder variants

For genes without known founder variants, we looked for truncating (except for *POLE* and *POLD1*) and missense variants that are 1) rare in general population using gnomAD (AF < 0.01); 2) predicted to be pathogenic by >3/9 prediction tools (SIFT, Polyphen 2 HVAR, LRT, MutationTaster, Mutation Assessor, FATHMM, RadialSVM, LR and with CADD phred >24; 3) with a minimum alternate read count of 30 reads and variant allele frequency (VaF) > 9%. We specifically looked for variants occurring more than once in our case series and present in the COSMIC database. Each variant was then verified manually by Integrative Genome Viewer (IGV).

### NGS Sanger validation of variants of interest identified by NGS

Where sufficient DNA was available, we used Sanger sequencing to validate all variants found in this study by custom amplicon sequencing using PCR amplification with different primers flanking the variant region.

## Results

Our bioinformatic pipeline analysis of the custom amplicon sequencing data identified 126 variants of interest (86 in *APC*, 11 in *POLE*, 11 *NTHL1*, 5 in *POLD1*, 5 *BLM*, 4 in *MSH2*, 2 in *GREM1*, 2 in *MSH6*). None of the variants detected in *POLE*, *POLD1* and *NTHL1* were confirmed by Sanger analysis, bringing the number of detected variants to 99 from 765 individuals (13%).

**Fig. 1:**
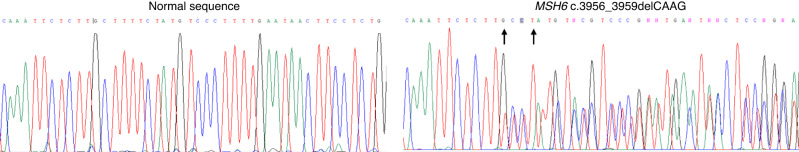
MSH6 c.3956_3959delCAAG frameshift deletion confirmed by Sanger sequencing found in both MM-65 and MM-72 cases. Arrows represent the beginning and end of the deletion.

Germline pathogenic variants found in *APC*, *MSH2*, *BLM* and *GREM1* were known in our case series from previous studies. We found two *MSH6* c.3959_3962delCAAG; p.(Ala1320Glufs*6) pathogenic variants that were confirmed by Sanger sequencing (Fig. 1). This frameshift variant leads to the creation of a stop codon downstream.

Variant frequency of *GREM1* 40 kb upstream duplication, *BLM* c.2207_2212delinsTAGATTC; p.(Tyr736Leufs*5), *APC* c.3920 T > A; p.(Ile1307Lys), *MSH6* c.3959_3962delCAAG; p.(Ala1320Glufs*6) and *MSH6* c.3984_3987dup; p.(Leu1330Valfs*12), *MSH2* c.1906G>C; p.(Ala636Pro) present in our study were in the same range as reported previously (Supplementary Table 1). We observed that our CO-control series could be considered “super-controls’ as we did not identify any positive cases in that series as compared to other previously published control series (95%CI[0.00, 0.022] *vs* 95%[0.066,0.075]). This finding supports the idea that persons with negative colonoscopy are less likely to be *APC* I1307K positive than controls who have not necessarily had this procedure; they are just not known to have colorectal neoplasia.

We next compared the frequency of each variant for all cases with tumors (CPC cases plus CO-p cases) as compared to controls from both our series (CO) and from published studies. (Table 2). Other than for the *APC* I1307K and the already known *MSH2* p.(Ala636Pro), we did not find any evidence for enrichment between cases and controls for the rest of variants, confirming the dominant role of this *APC* variant and *MSH2* in CRC and/or polyps predisposition. However, when we looked at whether patients presented with polyps only or were diagnosed with CRC with or without polyps, we did not find any differences in the prevalence of *APC* I1307K between these two groups of affected persons. On the other hand, despite the small numbers it is notable that the *MSH2* variant is more prevalent in those with CRC with or without polyps (3/103, 2.91%) than in those with only polyps (1/493, 0.2%), P value for difference 0.0176) (Table 3). This is consistent with older data suggesting that Lynch syndrome patients are not much more susceptible to polyps than are the general population, but when a polyp does arise, it is more prone to advance to a cancer than is the case in those without Lynch syndrome [24].

**Table 2:** Comparison of founder germline pathogenic variants frequencies for the variants studied between cases with tumors (CRC and CO-p series) and cases without tumors from our series (CO-c) and from the literature, limited to the Ashkenazi Jewish population.

		Positive Cases (CPC+CO-p)		Negative Cases												
				*APC Zauber - 2008 Valle - 2023* (18,24)		*BLM Zauber – 2008* (18)		*GREM1 Laitman – 2015* (20)		*MSH2 Foulkes - 2002 Zauber – 2008* (10,18)		*MSH6del Raskin – 2011* (12)		*MSH6dup Raskin – 2011* (12)		
		(*n*=600)		(*n*=13238)		(*n*=293)		(*n*=312)		(*n*=859)		(*n*=3475)		(*n*=3475)		*(p-value)*
**Gene**	**Variant**	**Frequency**														
*APC*	*c.3920T>A, p.(Ile1307Lys)*	86	14,33%	939	7,09%											(< 0.00001)
*BLM*	c.2207_2212delinsTAGATTC, p.(Tyr736Leufs*5)	5	0,83%			0	0,00%									(0.1785)
*GREM*	*40kb upstream duplication*	2	0,33%					0	0,00%							(0.5494)
*MSH2*	*c.1906G>C, p.(Ala636Pro)*	4	0,67%							1	0,12%					(0.0284)
*MSH6*	c.3956_3959delAAGC, p.(Ala1320Glufs*6)	1	0,17%									1	0,03%			(0.2728)
*MSH6*	c.3984_3987dupGTCA, p.(Leu1330Valfs*12)	0	0,00%											1	0,03%	(1)

CPC Colorectal and Polyps Cancer series, CO-p patient from Colonoscopy series, CO-c control from Colonoscopy series. Statistical test used is Fisher’s exact test.

**Table 3:** Comparison of studied germline pathogenic variants frequencies between patients with only polyps and patients with both polyps and cancer from the series we studied.

		CPC series					CO-p series					CPC+CO-p series				
		Polyps Only		Cancer & Polyps			Polyps Only		Cancer & Polyps			Polyps Only		Cancer & Polyps		
		357		77		*(p value)*	136		26		*(p value)*	493		103		*(p-value)*
**Gene**	**Variant**	**Frequency**														
*APC*	*c.3920T>A, p.(Ile1307Lys)*	41	11,48%	13	16,88%	(0.2526)	25	18,38%	5	19,23%	*NA*	66	13,39%	18	17,48%	(0.2779)
*BLM*	c.2207_2212deinsTAGATTC, p.(Tyr736Leufs*5)	4	1,12%	0	0,00%	*NA*	1	0,74%	0	0,00%	*NA*	5	1,01%	0	0,00%	(0.59)
*GREM*	*40kb upstream duplication*	2	0,56%	0	0,00%	*NA*	0	0,00%	0	0,00%	*NA*	2	0,41%	0	0,00%	*NA*
*MSH2*	*c.1906G>C, p.(Ala636Pro)*	0	0,00%	3	3,90%	(0.0059)	1	0,74%	0	0,00%	*NA*	1	0,20%	3	2,91%	(0.0176)
*MSH6*	c.3956_3959delAAGC, p.(Ala1320Glufs*6)	0	0,00%	0	0,00%	*NA*	0	0,00%	1	3,85%	(0.1667)	0	0,00%	1	0,97%	(0.1728)
*MSH6*	c.3984_3987dupGTCA, p.(Leu1330Valfs*12)	0	0,00%	0	0,00%	*NA*	0	0,00%	0	0,00%	*NA*	0	0,00%	0	0,00%	*NA*

CPC Colorectal and Polyps Cancer series, CO-p patient from Colonoscopy series, CO-c control from Colonoscopy series. Statistical test used is Fisher’s exact test. NA - not applicable.

**Fig. 2:**
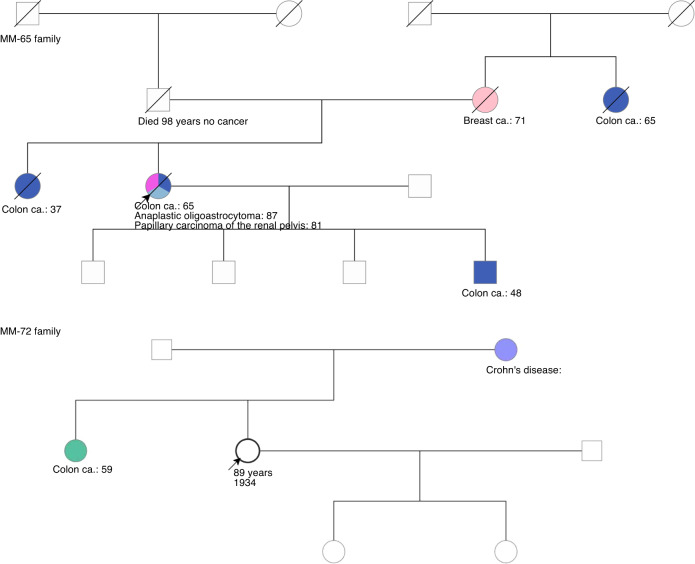
MM-65 and MM-72 MSH6 families pedigrees. Arrows represent index cases we tested in this study.

The *MSH6* GPV was observed in two women from unrelated families (the MM-65 and MM-72 families, Fig. 2). One woman had a strong personal history of colon and other cancers, whereas the other did not. The proband of MM-65 had colon cancer at age 65 diagnosed at another facility. Sixteen years later she had a nephrectomy for the removal of a papillary carcinoma of the renal pelvis, with clear margins. At age 82 years, she underwent resection of the splenic flexure and rectum, and two new primary cancers were found: an invasive adenocarcinoma of the splenic flexure and a second in the rectosigmoid with residual adenoma. Staging was pT3, pN0, pMx. Two years later, she underwent residual colectomy and removal of part of the small bowel. At that time a new colon cancer was found near the juncture of the small and large bowel. Two lymph nodes were negative for cancer. Three years later, she underwent a craniotomy for an anaplastic oligoastrocytoma, grade 3; this prompted us to look for the following known germline variants, which were negative: *APC* I1307K, *BLM*^*Ash*^, *MSH2* 1906G > C. Both colorectal tumors (the splenic flexure cancer and the rectosigmoid cancer) showed microsatellite instability. The splenic flexure tumor was also *KRAS* mutated, but the cancer in the rectosigmoid was not. Three family members were affected by colon cancer, including two that occurred at an early age. The proband of MM-72 had several colonoscopies and never had colon polyps or colorectal cancer, but her sister received a diagnosis of CRC at age 59 (Fig. 2).

## Discussion

We systematically sequenced 765 DNA samples at selected loci where CRC predisposition genetic variants might be detected in AJ persons undergoing at least one colonoscopy. The I1307K allele was found at a higher frequency in the CO-patient series (25/136 – 18.52%) as compared to CO-control (0/165) and CPC-series (56/438 – 12.79%). This difference can be explained by the smaller size of the CO-patient series, but also by the fact that *APC* I1307K frequencies increase with family history of CRC. Thus, *APC* I1307K frequencies are in the range of what was seen in previous studies: between 6%–10% of the AJ population and in ~15%–28% of AJ individuals with family history of CRC [8, 11, 18, 19, 25, 26]. In contrast, all other AJ founder GPVs were rare, albeit at slightly different frequencies than previously reported.

In the two *MSH6* cases, the discrepancy between the families can potentially be explained by the variable but generally low penetrance of *MSH6* in colorectal cancer [27]. Additionally, for some families, a second germline mutation in a critical gene, and/or a strong environmental factor may be required for tumor development. [28, 29] No endometrial cancer was observed in either family, whereas the penetrance has been reported to be approximately 50% for this gene [27]. We hypothesize that MM-65 mother’s breast cancer could be attributable to *MSH6*, since this gene has been associated with breast cancer predisposition [30, 31], but this cannot be tested.

It is noteworthy that the frequencies seen with our community population are similar to frequencies observed in more highly selected populations, as might be seen in referral centers. Also, we did not identify new founder pathogenic variants in the more recently described polyposis predisposition genes *POLE*, *POLD1* or *NTHL1*. In the general population, GPVs in those genes are known to be very rare, and it seems to be the same in selected AJ populations [32, 33]. From our data, the likelihood of the existence of a founder GPV in these proof-reading genes or in *NTHL1* in a community-based AJ population appears very low.

It remains possible that colorectal/polyposis founder pathogenic variants may be located in other putative predisposition genes like *RNF43* [34], or in other syndromic cancer predisposition syndromes associated with polyposis genes such as *PTEN*, *MBD4, SMAD4, STK11 or BMPR1A*. However, our focus on colorectal adenomas, rather than other types of colorectal polyps, makes this unlikely. Environmental factors such as smoking, obesity, and diet remain important influences for the development of colorectal neoplasms.

In a study of the AJ population, Rosner et al. reported 11 patients (from 132 = 8.3%) with multiple colorectal adenomas or colorectal cancer carrying either *POLE* or *POLD1* variants [35]. Eight of these 11 patients carried the same *POLD1* variant (V759I). This variant is located outside the exonuclease domain, a region not covered by our custom amplicon targeted sequencing. Moreover, the authors suggested the V7591 variant is a low-to-moderate risk founder GPV, but this variant has a frequency of 0,16% in the general population (gnomAD) and almost 2% in the AJ population, including four homozygous healthy subjects (gnomAD), making it more likely this variant is a polymorphism rather than a true pathogenic variant.

The presence of *APC* c.3920 T > A ; p.(I1307K) is associated with A8 slippage and it gives a modest non-early onset colorectal tumor risk (estimated to be 1.68-fold by meta-analysis) [25]. Those results are in line with a high previously reported *APC* I1307K frequency of approximately 10% in the AJ population and now in this study [19]. As Zauber et al demonstrated in a previous study of 82 colonic neoplasms from this case series, *APC*I1307K is associated with A8 slippage, but most of the observed *APC* hits are not related to this slippage [19]. Although this variant was found in patients with multiple adenoma or polyps, it was not associated with an increasing risk of polyposis [25]. A review of the literature by the InSiGHT consortium found no association between *APC* I1307K and extra-colonic cancer risk; one study found an association with melanoma and renal cancer [25, 36]. Notably, this variant is found in non-AJ persons but at a lower frequency, and shows no significant association with CRC [25, 37]. Thus, we consider it warranted to offer colonoscopy screening for AJ *APC* I1307K carriers with or without family history, as recommended by the InSiGHT consortium [25].

In summary, the study revealed that approximately 10 to 15% of Ashkenazi Jewish individuals who underwent colonoscopy carried a noteworthy GPV within well-established colorectal cancer and/or polyposis susceptibility genes. Conversely, it appears that GPVs within recently discovered genes linked to predisposition to polyps are infrequently encountered. The strength of this study is its large sample size of ethnically restricted patients all assessed with the same protocol at the same institution. The main challenge of the study is that many older samples had limited quantity and quality, which made variant discovery more difficult. The findings reported here highlight the significance of genetic testing, particularly in the Ashkenazi Jewish population where the frequency of a disease-associated variant can be higher than the general population, allowing for early detection and targeted interventions to mitigate the impact of this disease.

## Supplementary information

Supplementary information

Supplementary File

## Data Availability

Our code is available in Supplementary File 1. Data are available upon request to Dr William D. Foulkes.

## Data availability

Our data is available in Supplementary File 1. Data are available upon request to Dr William D.Foulkes.
